# Evaluation of existence and transmission of extended spectrum beta lactamase producing bacteria from post-delivery women to neonates at Bugando Medical Center, Mwanza-Tanzania

**DOI:** 10.1186/1756-0500-7-279

**Published:** 2014-05-03

**Authors:** Edwin Nelson, Juma Kayega, Jeremiah Seni, Martha F Mushi, Benson R Kidenya, Adolfine Hokororo, Antke Zuechner, Albert Kihunrwa, Stephen E Mshana

**Affiliations:** 1 Department of Microbiology/Immunology, Catholic University of Health and Allied Sciences, P.O. Box 1464, Mwanza, Tanzania; 2 Department of Biochemistry and Molecular Biology, Catholic University of Health and Allied Sciences, P.O. Box 1464, Mwanza, Tanzania; 3 Department of Pediatrics and Child Health, Catholic University of Health and Allied Sciences, P.O. Box 1464, Mwanza, Tanzania; 4 Department of Obstetrics and Gynaecology, Catholic University of Health and Allied Sciences, P.O. Box 1464, Mwanza, Tanzania

**Keywords:** ESBL, Post-delivery women, Neonates, Tanzania

## Abstract

**Background:**

Extended spectrum beta-lactamase producing bacteria (ESBL) are common causes of neonatal sepsis worldwide. Neonatal sepsis due to ESBL is associated with increased morbidity and mortality at Bugando Medical Centre (BMC). Due to limited information on the sources of these ESBL strains at BMC, this study was conducted to evaluate the existence, magnitude and transmission of ESBL from post-delivery women to neonates at BMC, Mwanza-Tanzania.

**Results:**

A cross-sectional study was conducted at obstetrics and neonatal wards from May to July 2013, involving post-delivery women and their neonates. Rectal swabs were collected and processed to identify the ESBL strains and their antimicrobial susceptibility patterns. Patients’ data were obtained using a standardized data collection tool. We enrolled 113 women and 126 neonates with mean age of 26.5 ± 5.5 years and median gestation age [IQR] of 39 [35–40] weeks respectively. The prevalence of ESBL carriage among women and neonates were 15% (17/113) and 25.4% (32/126) respectively. The acquisition of ESBL isolates among neonates on day 1, day 3 and day 7 were 60.0% (21/35), 25.7% (9/35) and 14.3% (5/35) respectively. There was no phenotypic similarity between ESBL strains from women and their respective neonates, suggesting other sources of transmission. Neonates given antibiotics were more likely to carry ESBL than those not given [100% (32/32) versus 86% (81/94), p = 0.018].

**Conclusion:**

The carriage rate of ESBL strains among post-delivery women and neonates at BMC is high. Our findings suggest that neonates acquire these strains from sources other than post-delivery women and more than half acquire them on the first day of life. More studies are recommended to further explore the sources of ESBL strains among neonates.

## Background

The escalating burden of multidrug resistant (MDR) gram negative bacteria of the family Enterobacteriaceae is currently one of the most challenging situations both in developed and developing countries [1-4]. The exposure of these bacteria notably, *Escherichia coli* and *Klebsiella pneumoniae* to antibiotics especially in the hospital environment has induced dynamic and continuous mutation leading to production extended spectrum beta lactamase (ESBL) which confer resistance to first, second, third and fourth generation cephalosporins as well as aztreonam [5-7]. These ESBL strains in turn have spread not only in the hospital environment but also to the community settings with significant regional variations [4,6,8,9].

Although shown in few studies, gastrointestinal carriage of ESBL strains can be a predictor of subsequent infection [10-12]. The prevalence of ESBL carriage has been shown to range from 3.2% to 67.9% [12-16]. Furthermore, pregnant women colonized by ESBL in the gastrointestinal tract have a potential to subsequently transmit these strains to their neonates (during and after delivery), to other hospitalized patients and to health workers [17,18].

Few studies have addressed the magnitude of ESBL associated infections with undue complications and deaths in Tanzania [3,9,19-21] however information showing the ESBL carriage among post-delivery women and their neonates remain to be explored. Thus, this study aimed at evaluating the existence, magnitude and transmission of ESBL strains from post-delivery women to their neonates during or after delivery so as to specifically guide infection prevention and control at Bugando Medical Center (BMC) where ESBL associated infections have been shown to be an independent predictor of deaths among neonates [3].

## Methods

### Study design and sampling procedures

This was a cross sectional hospital based study conducted from May to July 2013 in the obstetrics wards, neonatal ICU and neonatal wards at BMC. Post-delivery women who delivered at BMC within 24 hours and who consented to participate in the study were serially recruited and their respective neonates. The sample size of 113 post-delivery women was estimated using Kish Leslie (1965) formula and using a previous prevalence of 8% [14].

### Data and clinical sample collection

Every post-delivery woman was informed about details regarding the aims of the study. A standardized data collection tool and patients’ files were used to collect demographic and clinical data among consented women and their respective neonates.

Then, after a thorough explanation, using sterile cotton swab in Amies transport medium (Biolab, HUNGARY) rectal swabs were collected from women within 24 hours after delivery. To assess acquisition of ESBL strains in neonates, the rectal swabs were collected on day 1, day 3 and day 7 post-delivery. Rectal swab specimens were delivered to the Laboratory within two hours after collection.

### Laboratory procedures

The rectal swab samples were plated onto MacConkey agar (OXOID, Basingstoke, UK) supplemented with cefotaxime 2 mg/L for preliminary screening of ESBL bacterial isolates and then plates were incubated at 35-37°C for 18–24 hours. All gram negative bacteria were identified using phenotypic characteristics such as lactose fermentation reaction on Mac Conkey agar, Urease, Citrate, Sulphur indole motility (SIM) and Triple sugar iron (TSI) tests as previously described [22].

Susceptibility testing was done using disc diffusion method based on the Clinical Laboratory Standard Institute (CLSI) guideline [23]. The antibiotic discs tested were ampicillin (10 μg), amoxicillin/clavulanate (20/10 μg), ciprofloxacin (5 μg), tetracycline (30 μg), gentamicin (10 μg), sulfamethoxazole/trimethoprim (1.25/23.75 μg), ceftriaxone (30 μg), ceftazidime (30 μg) and imipenem (10 μg) (OXOID, Basingstoke, UK). The phenotypic confirmation of ESBL isolates was done using disc approximation method i.e. ceftazidime (30 μg) and cefotaxime (30 μg) discs were placed equidistant from the amoxicillin/clavunate (20/10 μg) disc, followed by overnight incubation at 37°C for 18–24 hrs. Enhanced zone of inhibition towards amoxicillin/clavulanate disc was considered as positive result for ESBL production whereas interpretation of susceptibility patterns on other antimicrobial disks was done using guidelines laid down in the CLSI, which provides break points corresponding to zone of inhibition diameter [9,23].

### Quality control

Standard laboratory procedures were strictly adhered to so as to avoid contamination. *Escherichia coli* ATCC 25922 and *Escherichia coli* ATCC 35218 were used as negative and positive ESBL controls respectively.

### Data management and analysis

The demographic, clinical and laboratory data were entered into Microsoft excel and then exported to the STATA version 11.0 software for analysis according to the objectives of the study. Results were presented into percentages/proportions for categorical variables whereas continuous variables were described as mean (±standard deviation). The difference in distribution of predictor variable was considered significant if p-value was less than 0.05.

### Study clearance and ethical consideration

The study was approved by CUHAS/BMC Ethical Review Board, and written informed consent was obtained from every post-delivery woman (who also provided consent for her neonate) before collection of demographic, clinical data and rectal swab specimens.

## Results

A total of 113 post-delivery women and 126 neonates with mean age of 26.5 ± 5.5 years and median gestation age [IQR] of 39 [35–40] weeks respectively were recruited.

**Figure 1:**
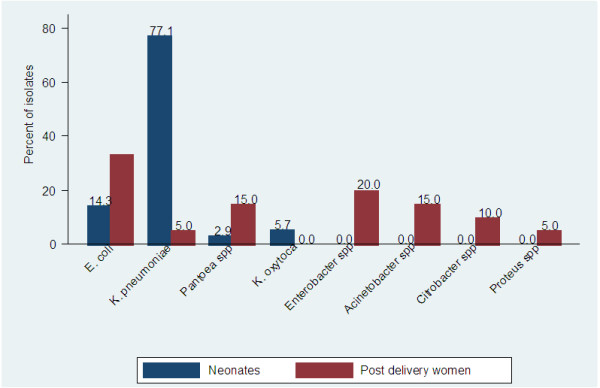
The proportion of ESBL bacterial isolates’ carriage among post-delivery women and neonates at BMC.

The prevalence of ESBL carriage among post-delivery women and neonates were 15% (17/113) and 25.4% (32/126) respectively. The cumulative number of ESBL bacterial isolates among post-delivery women and neonates were found to be 20 and 35 respectively throughout the study (three post-delivery women and neonates had dual ESBL carriage). The most predominant ESBL isolates among post-delivery women were *Escherichia coli* (30%, 6/20) followed by *Enterobacter spp* (20%, 4/20) whereas the predominant species in neonates were *Klebsiella pneumoniae* (77.1%, 27/35) followed by *Escherichia coli* (14.3%, 5/35) (Figure 1).

The acquisition of ESBL isolates among neonates on day 1, day 3 and day 7 were 60.0% (21/35), 25.7% (9/35) and 14.3% (5/35) respectively. Furthermore, there was no phenotypic similarity between ESBL strains from post-delivery women and their respective neonates.

There was no statistical significant association between ESBL-carriage among post-delivery women and variables such as age, mode of delivery, history of antibiotic use in the past or current admission, the presence of i/v line, urinary catheter insitu as well as HIV seropositivity. Neonates given antibiotics were more likely to carry ESBL than those not given [100% (32/32) versus 86% (81/94), p = 0.018]. The mortality rates among neonates carrying ESBL was relatively higher as opposed to non-carriers though the difference was not statistically significant (12.5%, 5/32 versus 9.6%, 9/94 respectively; p-value = 0.638) (Tables 1 & 2).

**Table 1:** Association between ESBL carriage among post-delivery women with variables

**Variable**	**Non ESBL carrier, n (%)**		**ESBL carrier, n (%)**	**p-value**
	**N = 96**		**N = 17**	
**Mean age ± SD**^**ǂ**^		27.1 ± 5.5 years	23.4 ± 3.6 years	0.084
**Mode of delivery**	C/S*	36 (37.5)	8 (47.1)	0.456
	SVD**	60 (62.5)	9 (52.9)	
**Admission in the past 30 days**	No	96 (100)	17 (100)	-
	Yes	0 (0.0)	0 (0.0)	
**Admission in the past 3 months**	No	83 (86.5)	13 (76.5)	0.288
	Yes	13 (13.5)	4 (23.5)	
**Previous use of antibiotics in the past 30 days**	No	67 (69.8)	9 (52.9)	0.172
	Yes	49 (30.2)	8 (47.1)	
**Previous use of antibiotics in the past 3 months**	No	54 (56.3)	7 (41.2)	0.250
	Yes	42 (43.7)	10 (58.8)	
**Antibiotics use in the current admission**	No	57 (59.4)	9 (52.9)	0.620
	Yes	39 (40.6)	8 (47.1)	
**Presence of catheter**	No	59 (61.5)	10 (58.8)	0.837
	Yes	37 (38.5)	7 (41.2)	
**Presence of i/v line**	No	54 (56.3)	10 (58.8)	0.844
	Yes	42 (43.7)	7 (41.2)	
**HIV serostatus**	Negative	84 (87.5)	16 (94.1)	0.431
	Positive	12 (12.5)	1 (5.9)	

^ǂ^Standard deviation; *Caesarean section; **Spontaneous vaginal delivery.

**Table 2:** Association between ESBL carriage among neonates with variables

**Variable**		**Non ESBL carrier, n**	**ESBL carrier, n**	**p-value**
		**N = 94**	**N = 32**	
**Median gestational (IQR)**^ǂ^		39 (35–40)	39 (35–40)	0.710
**Median birth weight (kg)**		2.6 (1.9-3)	2.9 (1.7-3.4)	0.365
**Sex**	Female	44 (46.8%)	17 (53.1%)	0.537
	Male	50 (53.2%)	15 (46.9%)	
**Mode of Delivery**	C/S*	35 (37.2%)	12 (37.5%)	0.979
	SVD**	59 (62.8%)	20 (62.5%)	
**Ward**	NICU^×^	19 (20.2%)	8 (25.0%)	0.569
	C2NU^××^	75 (79.8%)	24 (75.0%)	
**Antibiotic Use**	No	13 (13.8%)	0 (0.0%)	0.018
	Yes	81 (86.2%)	32 (100.0%)	
**Outcome**	Discharged	85 (90.4%)	28 (87.5%)	0.638
	Died	9 (9.6%)	4 (12.5%)	

^ǂ^Interquantile range; *Caesarian section; **Spontaneous vagina delivery; ^×^Neonatal intensive care unit; ^××^Ward C2 neonatal unit.

The ESBL bacterial isolates in this study showed borderline resistance to ciprofloxacin among post-delivery women (55.0%, 11/20) and neonates (34.3%, 13/35). All ESBL strains were sensitive to imipenem (Table 3).

**Table 3:** Antimicrobial resistance patterns among ESBL isolates from post-delivery women and neonates

**Antimicrobial agents**	**Post-delivery women, n (%)**	**Neonates, n (%)**
	**N = 20**	**N = 35**
Co-trimoxazole	20 (100.0)	25 (71.4)
Tetracycline	14 (70.0)	17 (48.6)
Gentamicin	15 (75.0)	32 (91.4)
Ciprofloxacin	11 (55.0)	12 (34.3)
Imipenem	0 (0.0)	0 (0.0)

## Discussion

The prevalence of ESBL carriage among women shown in this study (15%) is relatively close to 10% to 12.7% found in some Asian and African countries [13,15,16] but lower than 3% and 3.2% in Sweden and USA respectively [11,24]. The lower rates in ESBL carriage in developed countries as opposed to developing countries may be attributable to different policies in antimicrobial use and infection control in the respective settings. The present study and a similar one in Madagascar found the rates of ESBL carriage in children to be 15% and 22.1% respectively, with acquisition increasing progressively in the course of hospitalization [25]. This emphasizes the need to protect this vulnerable population of children against the MDR strains in the hospital settings. The predominance of *Escherichia coli* and *Klebsiella pneumonia* producing ESBL strains in this study is also similar to findings in some other studies across the world showing their preponderance in gastrointestinal tract colonization and their evolutionary potential into MDR strains [12,15,25,26].

It is well known that gastrointestinal carriage of ESBL strains among pregnant women can be a potential source of transmitting these resistant strains to their newborns [17,18] but contrary to this, there was no phenotypic similarity between ESBL strains from women and their respective neonates. This suggests that effective control of transmission of ESBL strains at BMC should go beyond mothers and that other possible sources should be further scrutinized so as to curb the growing burden of antimicrobial resistance in Tanzania [3,20] and other countries with similar problem [4,7].

As shown in some other studies [25-29], previous use of antibiotics among neonates has also been shown to be associated with ESBL carriage in the present study. The possible reason of high neonatal ESBL carriage in our setting may be due to antimicrobial selective pressure conferred by the empirical use of ampiclox and cefotaxime. Therefore, as shown from other settings [11], neonatal screening for ESBL strains and adherence to the rational antimicrobial use policy should be an enduring exercise at BMC. Other predictive factors such as older age, prolonged hospitalization, vaginal delivery, low birth weight and prematurity though shown in other studies [14,25,26,30] were not found to be statistically associated with ESBL carriage in the present study.

The MDR nature of the ESBL isolates in the present study is worrisome as these post-delivery women and their neonates may be the potential sources of these strains in the hospital environment, and should of necessity be among the target groups in infection prevention and control activities at BMC. The present study has shown excellent activity of carbapenems and borderline activity of ciprofloxacin against ESBL strains, the findings which are similar to other studies [8,25].

## Conclusion

The carriage rates of ESBL strains among neonates and their mothers at BMC are high. Our findings suggest that neonates acquire these strains from sources other than post-delivery women and more than half acquire them on the first day of life. These two groups should be among the target groups for ESBL screening at BMC so as to prevent ESBL spread in the hospital environment. In the light of these findings, more studies are recommended to further explore the sources of the ESBL strains among neonates.

## Competing interests

Authors declare that they have no competing interests.

## Authors’ contributions

Conceived, designed and executed the study: EN, JK, JS, MFM, BRK and SEM; Management of post-delivery women and neonates: AH, AZ and AK; Laboratory analysis of samples: EN, JK, JS, MFM and SEM; Data collection and analysis: EN, JK, JS and BRK. Wrote the initial draft of the manuscript: EN, JS and JK which was critically revised by all authors. All authors read and approved the final manuscript.
